# The Engineered Antibiotic Peptide PLG0206 Eliminates Biofilms and Is a Potential Treatment for Periprosthetic Joint Infections

**DOI:** 10.3390/antibiotics11010041

**Published:** 2021-12-30

**Authors:** David Huang, Nicholas Pachuda, John Michael Sauer, Dessie Dobbins, Jonathan Steckbeck

**Affiliations:** Peptilogics, 2730 Sidney Street, Suite 300, Pittsburgh, PA 15203, USA; nicholas.pachuda@peptilogics.com (N.P.); johnmichael.sauer@peptilogics.com (J.M.S.); dessie.dobbins@peptilogics.com (D.D.); jonathan.steckbeck@peptilogics.com (J.S.)

**Keywords:** periprosthetic joint infections, antimicrobial peptides, antibiotic resistance, multidrug resistance

## Abstract

Antimicrobial peptides (AMPs) have recently gained attention for their potential to treat diseases related to bacterial and viral infections, as many traditional antimicrobial drugs have reduced efficacy in treating these infections due to the increased prevalence of drug-resistant pathogens. PLG0206, an engineered cationic antibiotic peptide that is 24 residues long, has been designed to address some limitations of other natural AMPs, such as toxicity and limited activity due to pH and ion concentrations. Nonclinical studies have shown that PLG0206 is highly selective for targeting bacterial cells and is not toxic to human blood cells. Antibiofilm experiments demonstrated that PLG0206 is effective at reducing both biotic and abiotic biofilm burdens following direct biofilm contact. PLG0206 has rapid and broad-spectrum activity against both Gram-positive and Gram-negative bacteria that are implicated as etiologic agents in periprosthetic joint infections, including multidrug-resistant ESKAPE pathogens and colistin-resistant isolates. A recent first-in-human study demonstrated that PLG0206 is well tolerated and safe as an intravenous infusion in healthy volunteers. Studies are planned to determine the efficacy of PLG0206 in patients for the treatment of periprosthetic joint infections. This review summarizes the chemistry, pharmacology, and microbiology of PLG0206 and explores its current preclinical, clinical, and regulatory status.

## 1. Introduction

Antibiotics have been used as successful treatments for many bacterial diseases, but the increasing prevalence of antibiotic-resistant microorganisms is a major public health concern. Antimicrobial peptides (AMPs) have gained much attention for their potential to treat diseases related to bacterial and viral infections, specifically against pathogens that have become multidrug resistant (MDR), extensively drug resistant (XDR), or pan-drug resistant (PDR) [1,2].

AMPs are a class of antimicrobial effector molecules of 10–50 amino acids in length that contain an amphipathic structure as a consensus motif and are found in many species across all kingdoms of life. As components of the mammalian innate immune system, they contribute to the first line of defense against invading pathogens [3,4]. Human cathelicidin (LL-37) broadly represents the class of AMPs and is the best characterized of the natural AMPs. LL-37 exhibits both antimicrobial activity and host immunoregulatory properties that are common among naturally occurring molecules [5]. However, AMPs have not demonstrated significant success therapeutically, primarily due to their toxicity when administered systemically and activity that is highly sensitive to pH and ion concentrations [3,6].

**Figure 1:**
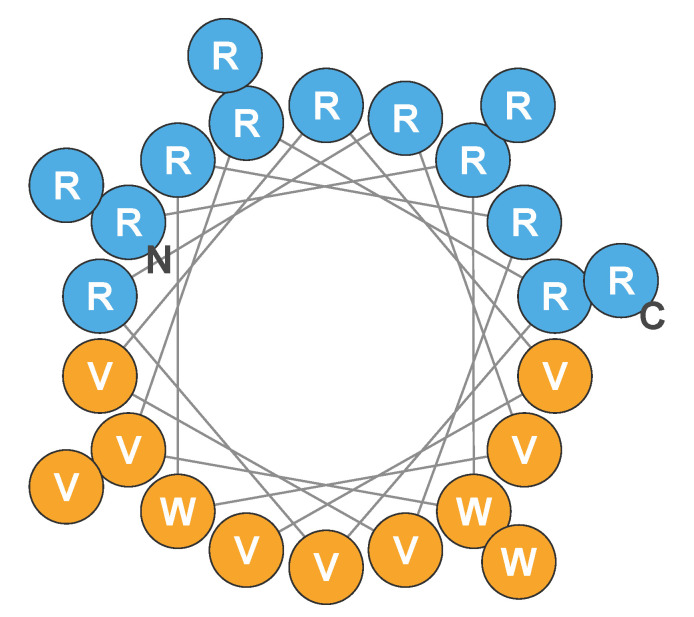
Helical wheel of the predicted structure of PLG0206. Blue circles indicate hydrophilic (cationic) amino acid residues; orange circles represent hydrophobic residues. C, carboxy terminus; N, amino terminus; R, arginine; V, valine; W, tryptophan. Adapted from Deslouches et al., 2005, 2015, and 2020 [9,10,11].

Engineered cationic antibiotic peptides (eCAPs) such as PLG0206—also known as WLBU2—have been designed to mitigate the shortcomings of natural AMPs and exhibit a broadened spectrum of activity, with increased activity in complex biological environments and an improved systemic safety profile [7]. PLG0206 is a 24-residue de novo eCAP [8]. PLG0206 has 13 arginine residues, 8 valine residues, and 3 tryptophan residues in the hydrophobic face that are separated from each other by at least seven amino acids (RRWVRRVRRWVRRVVRVVRRWVRR) [7]; the predicted structure is shown in Figure 1 [9,10,11].

Due to its antibacterial and antibiofilm activity, PLG0206 may have the unique potential to successfully address infections related to implanted medical devices, which are prone to biofilm-related infections that are difficult to eradicate. One of the potential applications for PLG0206 is treating infections in humans that occur after joint replacement. Periprosthetic joint infections (PJIs) following total knee arthroplasty and other types of joint replacements can be a major problem for patients, caregivers, and health care providers; these infections are potentially fatal [12]. PJIs have an estimated annual incidence of 2% in both total hip and total knee replacements [13]. These infections can occur at different intervals—early, delayed, and late onset—depending on the type of pathogen, and it is believed that more virulent microorganisms are responsible for early-onset PJIs that occur within 3 months after surgery. Prominent bacteria that cause early infections include *Staphylococcus aureus*, *Enterococcus* spp., and aerobic Gram-negative bacilli, as well as coagulase-negative staphylococci [14,15]. Approximately 31–36% of early infections are polymicrobial and may require broad-spectrum antimicrobial therapy [14,16].

When PJIs are diagnosed in the acute phase, their treatment includes debridement of the wound (i.e., irrigation and debridement, I&D) and 6 weeks of intravenous antibiotic therapy [17]. I&D has a high long-term treatment failure rate of approximately 57% at 4 years [12]. In most cases, I&D failure is followed by a two-stage revision and removal of the prosthesis in 54.1% of cases [12]. All options after treatment failure require surgical procedures with rehospitalization, immobility, and compromised daily functioning, resulting in a 20% risk of mortality within 5 years after an I&D procedure or the initial surgical intervention [12,18]. A major cause of failure of I&D is the presence of a biofilm, leading to bacterial “persisters” that result in extreme difficulty in eradicating the pathogen from the implant [19,20]. Cefazolin, rifampin, nafcillin, ceftriaxone, and vancomycin are commonly recommended antibiotics used for a 6-week intravenous treatment after I&D [17], but these antibiotics are inadequate to remove *S. aureus* biofilm from prostheses [19]. There are currently no drugs specifically indicated to disrupt biofilms or to target the “persister” pathogens in biofilms.

This review intended to provide an update of the chemistry, pharmacology, and microbiology of PLG0206 and to summarize the preclinical, clinical, and regulatory status of this eCAP for health care providers. It also will underscore the progress that has been made in administering AMPs safely to humans and using AMPs against biofilms and infections, including PJIs.

## 2. Pharmacology

### 2.1. Preclinical Pharmacology

#### 2.1.1. Mechanism of Action

Several studies have examined how PLG0206 exhibits bactericidal efficacy. Heinrich et al. [21] showed that neither the secondary structure nor pore formation is a critical determinant of bactericidal efficacy for PLG0206. The interaction of PLG0206 with the bacterial cell membrane leads to lipid phase consolidation, resulting in localized stiffening [22], ordering, and alteration of the thickness of the membranes [21]. Consequently, this disruption of the bacterial cell membrane results in lipid headgroup spacing mismatch and lowering of the energy barrier to ion flow across the membrane.

#### 2.1.2. Selectivity for Bacterial Cells

The selectivity of PLG0206 for bacterial membranes is driven, in part, by the electrostatic interaction between the cationic charge of the peptide and the net negatively charged bacterial membranes. The potential toxicity of PLG0206 to eukaryotic cells, which also carry a net negative charge, was investigated by co-culturing *Pseudomonas aeruginosa* with primary human skin fibroblasts [9], peripheral blood mononuclear cells [7], and red blood cells [23] in the presence of PLG0206. At the end of the incubations, killing of *P. aeruginosa* versus lysis of the human cells was determined. These studies demonstrated that PLG0206 had little to no cytotoxic effects on the primary human cells at concentrations that effectively killed *P. aeruginosa.*

In preclinical studies, PLG0206 in phosphate-buffered saline (PBS) was not toxic to human red blood cells (up to 20 µM) and demonstrated low hemolytic potential (up to 10 µM) [23]. PLG0206 in whole human blood also demonstrated no toxicity and low hemolytic potential (to 50 µM) [7]. These data provide encouraging evidence that PLG0206 is not toxic to human blood cells and may be a viable systemic treatment in humans.

#### 2.1.3. Degradation

The proteolytic activity associated with red blood cells is distinct from that of serum proteases and must be considered for any AMPs under development, as quicker degradation can result in loss of activity [24]. When incubated with cytosolic extracts of red blood cells, PLG0206 at 20 μM had a slow degradation profile and performed better than most other natural and synthetic peptides previously studied [24]. Furthermore, the robustness of intravenously administered PLG0206 to proteolytic degradation was demonstrated in a Phase 1 clinical study showing a median terminal half-life of up to 19.97 h [25].

#### 2.1.4. Pharmacokinetics and Absorption, Distribution, Metabolism, and Excretion

The absorption, distribution, metabolism, and excretion (ADME) of PLG0206 have been initially characterized. In human plasma, the plasma protein binding level of PLG0206 was determined to be ~98%, which is similar to the levels observed in animal plasma. Administration of radiolabeled PLG0206 in mice [26] showed rapid distribution to kidneys and spleen. Although the metabolism of PLG0206 has not been definitively evaluated, all tissues examined contained measurable PLG0206-derived ^14^C. The majority of an injected ^14^C dose recovered in non-carcass tissues was detected in the liver, kidney, and plasma at 5 min post-dose (32.8%, 9.0%, and 7.75%, respectively), 15 min post-dose (36.3%, 11.1%, 5.98%, respectively), and 24 h post-dose (8.6%, 2.6%, and 1.5%) [27]. The average recovery of ^14^C in the urine was 6.29% between 0 and 16 h and 12.5% between 0 and 24 h. The average recovery of ^14^C in the feces was 0.73% between 0 and 16 h and 2.2% between 0 and 24 h. Thus, ~2.3% of the dose was excreted over 24 h. Of the total ^14^C dose injected, the mean recovery was 106.6%, 99.4%, and 65.5% at 5 min, 15 min, and 24 h post-dose, respectively [26,27].

The pharmacokinetics of PLG0206 were consistent across animal models. Exposure, in terms of area under the plasma concentration time curve from time 0–*t* (AUC_[0–*t*]_) and the maximum plasma concentration (C_max_), increased with increasing dose in a greater than dose-proportional manner. The total plasma clearance of PLG0206 was considered moderate to high in animals, with a tendency to decrease clearance with an increasing dose. The calculated terminal elimination half-life of PLG0206 following a single dose was approximately 2 h in rats and 8 h in cynomolgus monkeys [26].

In a rabbit femorotibial joint debridement model, PLG0206 at 0.4, 1.2, and 3.6 mg in 0.9% saline was applied to the joint at a volume of 2 mL/animal (dose concentrations of 0.2, 0.6, and 1.8 mg/mL) for 30 min (twice the planned 15-min duration in human studies). Blood samples were taken pre-dose and up to 24.5 h after the start of exposure (SOE). Plasma PLG0206 concentrations were below the lower limit of quantitation (LLOQ; 5 ng/mL) in plasma samples at all time points from animals in the 0.4- and 1.2-mg groups. In the 3.6-mg group, PLG0206 was quantifiable for up to 2 h post-SOE, and the time to maximum plasma concentration (T_max_) was observed at 0.533 h post-SOE. The C_max_ was 9.30 ng/mL, and the AUC_0–24_._5_ was 9.68 ng*h/mL. Additionally, in a local administration minipig femorotibial joint debridement model, the animals underwent surgery, and the femorotibial joint was irrigated with a vehicle or PLG0206 at doses of 3, 10, and 30 mg/mL (15, 50, or 150 mg within 5 mL/animal). The mean C_max_ values for these doses were 2.30, 0.454, and 13.6 ng/mL, respectively [28]. Thus, systemic exposure was expected to be low in humans following irrigation with PLG0206.

A population pharmacokinetic model was developed from the observed PLG0206 pharmacokinetic data pooled from a total of nine animal studies (one in mice, three in rats, one in dogs, and four in monkeys). PLG0206’s pharmacokinetics were best described by a three-compartment zero-order absorption model. A population pharmacokinetic model showed that the bioavailability of PLG0206 increases with increasing weight (which is associated with the size of the species). This means that bioavailability is higher in larger species for the same milligram-per-kilogram dosage. Overall, the population pharmacokinetic model described the observed data in all species and was deemed appropriate for use in simulations of pharmacokinetic profiles in animals and extrapolation to humans using body weight (by allometric scaling). There was no effect of sex on pharmacokinetics observed in any of the species. Finally, based on the rabbit and minipig data presented above, it appears that systemic exposure following application of PLG0206 to surgically exposed femorotibial joint is minimal due to poor absorption from the space.

#### 2.1.5. Preclinical Safety

A comprehensive battery of in vitro and in vivo safety pharmacology studies has been completed for PLG0206. In an in vitro study in human embryonic kidney cells and Chinese hamster ovary cells, PLG0206 (0.1 μM) had no biologically meaningful effect on KvLQT1/mink and little to no effect on either hERG or Nav1.5 channel currents. In a cardiovascular assessment study, PLG0206 administered in an intravenous infusion (1 h) at doses of 3 and 6 mg/kg via a single dose and 15 mg/kg for 10 consecutive days to conscious cynomolgus monkeys was not associated with any changes in the electrocardiogram (PR, QRS, QT, QTcF). No effects of PLG0206 were observed on the respiratory system of rats following a single intravenous infusion (1 h) at a dose of ≤6 mg/kg body weight [28]. Overall, the available pharmacology data for PLG0206, including the safety data from cardiovascular, respiratory, and central nervous system function studies, support the development of PLG0206 as a therapeutic for PJI, where systemic exposure is expected to be very low.

PLG0206 was evaluated for the inhibition of radioligand binding to a broad panel of clinically important receptors, ion channels, and transporters and for inhibition of the activity of a panel of enzymes. At a concentration of 10 μM, PLG0206 exhibited >50% binding to a handful of receptors, channels, transporters, and enzymes. The functional activity of PLG0206 on these targets is currently being evaluated in secondary functional assays.

Importantly, all genetic toxicology studies for PLG0206 were negative, indicating a low genotoxic potential. In the nonclinical studies, PLG0206 was formulated in 0.9% sodium chloride, which is identical to the formulated clinical drug product for the first-in-human study.

To evaluate systemic safety, PLG0206 was administered to rats at doses of 0, 1, 3, and 6 mg/kg via 1-h infusions once daily for 14 days. In this study, PLG0206 appeared to be safe and well tolerated. In a different model, PLG0206 was administered once daily at 3, 6, and 12 mg/kg via a 1-h infusion for 14 days in cynomolgus monkeys, and PLG0206 was well tolerated [28]. No safety issues were noted in the femorotibial joint debridement studies in minipigs and rabbits when PLG0206 was applied to the joint for 30 min. Overall, systemic administration and joint application of PLG0206 were well tolerated and safe [28].

### 2.2. Human Pharmacokinetics

**Figure 2:**
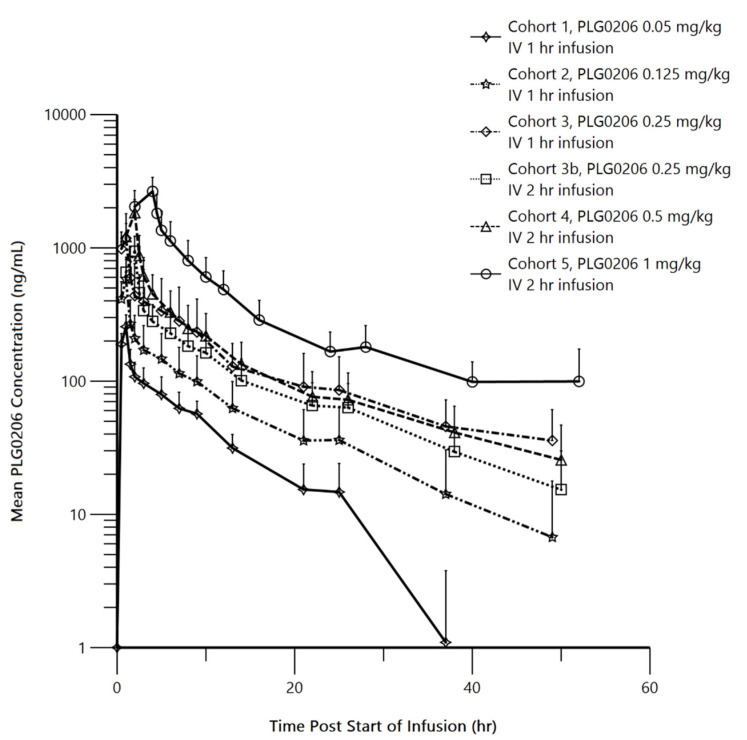
Mean (SD) PLG0206 plasma concentration (in log scale) time plots by dose group. IV, intravenous; hr, hour. Reproduced with permission from Huang et al., 2021 [25].

The pharmacokinetics of PLG0206 were evaluated in 33 healthy volunteers, as summarized in Figure 2. PLG0206 was administered intravenously and exhibited linear pharmacokinetics over the dose range of 0.05–1.0 mg/kg. The median terminal elimination half-life was 7.37–19.97 h [25]. The AUC_(0–*t*)_ ranged from 1283.74 h*ng/mL at 0.05 mg/kg to 12,612.56 h*ng/mL at 1 mg/kg. The AUC_(0–∞)_ was between 1581.41 h*ng/mL at 0.05 mg/kg and 21,141.52 h*ng/mL at 1 mg/kg. The C_max_ (mean ± SD) increased from 256 ± 58 ng/mL in Cohort 1 (0.05 mg/kg, intravenous, 1 h) to 2653 ± 719 ng/mL in Cohort 5 (1 mg/kg, intravenous, 4 h) [25]. The mean apparent volume of distribution increased from 25.49 L in Cohort 1 to 94.2 L in Cohort 5. The mean clearance values (range, 2.42–4.18 L/h) were similar across all PLG0206 doses and infusion times [25].

## 3. Microbiology

PLG0206 has demonstrated activity against >200 clinical isolates in vitro, ex vivo, and in vivo via various routes of administration. Table 1 below shows activity against coagulase-negative staphylococci and resistant Gram-negative pathogens. The following sections describe specific conditions that enhance its bactericidal activity against various pathogens.

In a reference panel of 142 clinical isolates, with 58% of these representing ESKAPE (*Enterococcus faecium*, *S. aureus*, *Klebsiella pneumoniae*, *Acinetobacter baumannii*, *P. aeruginosa*, and *Enterobacter* species) pathogens, PLG0206 had a mean minimum inhibitory concentration (MIC) of <10 μM against both Gram-positive and Gram-negative strains and was less likely to elicit resistance from these strains [11]. PLG0206 had a lower MIC and less resistance than LL-37 and colistin, and comparable activity with the eCAP WR12 against ESKAPE pathogens and XDR/MDR strains [11,23]. Moreover, PLG0206 exhibited activity that was much higher than that of colistin and LL-37 in the inhibition of biofilm growth by preventing bacterial attachment to solid surfaces (e.g., medical implants or surgical sites) [30]. By comparison, traditional antibiotics occasionally stimulated more bacterial attachment rather than preventing biofilm formation [30]. AMPs inhibited more biofilm at sub-MICs compared with traditional antibiotics, with only ceftazidime showing 25–50% prevention of biofilm formation at 1× MIC against *S. aureus*; all other antibiotics (tobramycin, ciprofloxacin, and vancomycin) showed negligible biofilm prevention activity against Gram-positive *E. faecium* and *S. aureus* [30]. Thus, identification of the AMPs that significantly prevent biofilm formation can be useful for the prevention of PJIs and other infections where biofilms are implicated in the pathogenesis.

### 3.1. PLG0206’s Antibiofilm Activity

Biofilms can develop in multiple tissues (e.g., urinary tract, kidney, lung, heart, eyes, sinuses) as well as on multiple devices (e.g., contact lenses, pacemakers, breast implants, orthopedic implants, and prosthetic joints) [31]. Microorganisms that grow into biofilms differ from those that grow planktonically, in that they have physiological and biochemical gradients from the surface to the deeper layers that are attached to the tissue/device [32]. Thus, the aggregated biofilm structure makes it difficult for antibiotics and the human immune system to reach the source of a chronic infection caused by the microorganism [31,32]. Areas of interest include how to help combat the development of biofilms and chronic infections in the airways of patients with cystic fibrosis and prosthetic joints.

PLG0206 significantly decreased *P. aeruginosa* biofilm growth on an abiotic surface (*p* < 0.05) and on airway epithelial cells under conditions similar to those in the lungs of patients with cystic fibrosis [33]. PLG0206 disrupted *P. aeruginosa* biofilms in a dose-dependent fashion, with ~90% biomass reduction at concentrations above 10 μM [34]. Likewise, on biofilms that formed on respiratory epithelial cells, PLG0206 reduced biomass by ~85% and reduced colony-forming units by approximately 50-fold [34]. This suggests that PLG0206 can be effective in disrupting biofilms in different physiological settings.

As mentioned previously, PJIs lead to increased mortality and decreased quality of life for patients who have joint replacements. Given PLG0206′s direct antibacterial and antibiofilm effects, evaluation of local administration as a surgical irrigation solution was pursued. The activity of PLG0206 was compared in buffered solutions commonly used for irrigation of infected prostheses removed from patients with a PJI in the operating room. PLG0206 demonstrated a greater efficacy at reducing biofilm mass on the implants’ surfaces at higher pH solutions and achieved over 99.9% reductions in bacterial biofilm at concentrations of 62–1000 μg/mL in lactated ringers and diphosphate-buffered saline (dPBS) (Table 2) [35]. Under alkaline conditions, PLG0206 exhibited a reduction in contact time for bactericidal activity; under physiologic pH conditions, PLG0206 achieved complete inhibition of bacterial growth on blood agar plates [35].

In an ex vivo murine model that analyzed the biofilm burden on implants, immersion in PLG0206 for 10 min at various pH levels showed a significant reduction in biofilm burden at pH 7.4 compared with the no-drug control group (*n* = 5; *p* = 0.005), which was also significant compared with PLG0206 immersion at pH 6.5 (*p* = 0.04) (Table 3) [35]. Overall, these in vitro and ex vivo experiments demonstrated that PLG0206 can be formulated in solutions that are used clinically and that the pH can be adjusted to decrease the needed contact time during local drug delivery [35].

Recent work on PLG0206 has documented that it is a rapidly acting and highly effective antibiofilm agent, in addition to its established activity against planktonic *Staphylococcus* (both in vitro and in a murine model of PJI [36]) and on biofilms produced by *S. aureus* (including methicillin-resistant strains). There is no evidence of cross-resistance for PLG0206 with established known mechanisms of resistance among the panel of currently circulating clinical isolates (e.g., methicillin-resistant *S. aureus*, vancomycin-resistant *Enterococcus*, MDR), as its activity against resistant isolates was consistent with its activity against susceptible isolates. In serial passage studies with three isolates of *P. aeruginosa*, resistance to PLG0206 was not observed until Passage 24 for two isolates and Passage 30 for the remaining isolate [11].

### 3.2. Antibacterial Activity of PLG0206 on Implant Surfaces

**Figure 3:**
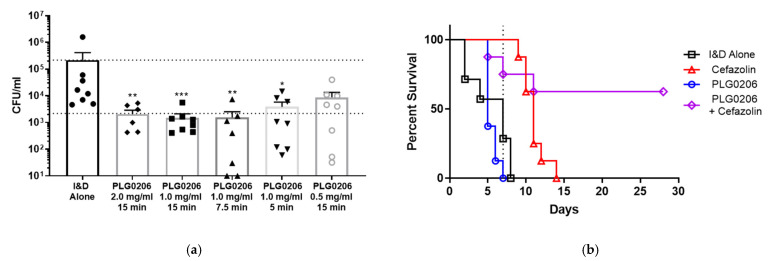
Activity of PLG0206 for treating *S. aureus* PJI in a large animal model. (**a**) In vivo intra-articular treatment of PJI with PLG0206 for 15 min reduced *S. aureus* biofilms. * *p* < 0.05, ** *p* < 0.01, *** *p* < 0.001. (**b**) Treatment with cefazolin systemically and PLG0206 intraoperatively resulted in increased survival. Data from Brothers et al., 2021 [38].

Two different models of PJI were developed in rabbits to distinguish between acute and chronic PJI and to determine the benefit of treatment with PLG0206 at different stages of infection. In a rabbit model of chronic PJI, implants treated 2 days post-infection with 1 or 2 mg/mL PLG0206 for 15 min produced a significant reduction in the bacterial biofilm burden of over 100-fold (*p* < 0.05; Figure 3a) [37,38]. In an associated survival study of rabbits treated with PLG0206, cefazolin, or both, improved survival was observed in rabbits treated with both—63% at 28 days post-infection (*p* < 0.05; Figure 3b)—whereas individual treatments of cefazolin alone led to 0% survival (PLG0206, 8 days; cefazolin, 14 days) [38]. In a separate study with an acute PJI rabbit model, ex vivo treatment with PLG0206 after 48 h resulted in a >3.3 log_10_ reduction in biofilm [39]. When PLG0206 was used as the irrigation adjuvant in an I&D procedure, survival was 63% compared with 0% (100% mortality) in the traditional I&D control group (*p* < 0.05) [39].

## 4. Clinical

PLG0206 has been evaluated in a Phase 1 trial in healthy volunteers to establish the safety and tolerability of intravenous infusions and to determine pharmacokinetic properties.

### 4.1. Safety

The results of multiple nonclinical studies indicated a safety profile of PLG0206 that supported the Phase 1 first-in-human clinical study in healthy volunteers. This is significant, as many AMPs have not advanced into clinical trials because of the toxicity observed in nonclinical studies. In a Phase 1 trial of 47 healthy volunteers, there was similar incidence of treatment-emergent adverse events related to PLG0206 between the treatment and placebo groups [25]. Most events were mild in severity. The most common adverse events were infusion-related reactions, which resolved with increasing infusion time and volume. PLG0206 demonstrated safety with no serious adverse events, no life-threatening events, and no deaths throughout the study [25]. This suggests that PLG0206 has an acceptable safety profile when administered intravenously in humans and can advance to proof-of-concept studies to determine efficacy and safety in a patient population.

### 4.2. PLG0206’s Activity in Ex Vivo Model of PJI

Another route of administering PLG0206 would be as a component in irrigation solutions for surgical implants. Therefore, patients were recruited to examine the extent of bacterial burden that PLG0206 could remove from infected implants. The surgically removed implants of patients with chronic PJI were rinsed and submerged ex vivo in 1 mg/mL PLG0206 in dPBS at pH 7.4 for 15 min, and the samples were rinsed, sonicated, and plated for quantitative culturing [40]. After this treatment, the bacterial burden showed more than 3-log reductions in both Gram-positive and Gram-negative bacteria [40]. Additionally, complete removal of bacteria was observed in 10 of 18 (56%) infected implants treated with PLG0206; there was a mean log reduction of 6 (range, 2.9–7.70) of the infected prosthetics exposed to PLG0206 [40]. These findings suggest that a concentration of at least 1 mg/mL PLG0206 in the local irrigation solution used in the wound cavity for 15 min would reduce the bacterial load for patients requiring treatment of a PJI [40].

## 5. Regulatory

PLG0206 gained qualified infectious disease product (QIDP) status from the US Food and Drug Administration in May 2018 for the treatment of PJIs, which provides expedited regulatory review periods and extended marketing exclusivity. Moreover, the orphan drug designation has been granted. The first Investigative New Drug (IND) application will be submitted in 2021 for an irrigation solution of PLG0206 for PJI, with plans for separate INDs for new formulations and indications in the future. A safety, tolerability, and pharmacokinetic trial is planned in the target population of patients with PJIs and will begin in 2022.

## 6. Conclusions and Summary

PLG0206 is being developed for the treatment of PJIs. PLG0206 is an eCAP, a synthetic antimicrobial agent based on the physicochemical properties of naturally occurring AMPs. Cumulative PLG0206 antibiofilm experiments performed to date have demonstrated that PLG0206 is effective at reducing both biotic and abiotic biofilm burdens following direct biofilm contact. PLG0206 has a rapid, broad spectrum of activity encompassing both Gram-positive and Gram-negative bacteria that are implicated as causative agents in PJI, including resistant and MDR ESKAPE pathogens and colistin-resistant isolates. A comprehensive program of nonclinical safety studies has been undertaken to characterize the safety profile of PLG0206, which supported a Phase 1 intravenous clinical study in healthy volunteers. The results from the first-in-human Phase 1 study supported advancing the program with a safety/pharmacokinetic Phase 1b study using PLG0206 as an irrigation solution in patients with PJI. The results of this study will be used to support further development for Food and Drug Administration review and approval as an irrigation solution for orthopedic use in PJIs.

## Figures and Tables

**Table 1:** Activity of PLG0206 and comparable products against coagulase-negative staphylococci and resistant Gram-negative pathogens. Data from Murray et al., 2020 [29].

Organism	Drug	MIC Range, µg/mL
All *Staphylococcus epidermidis* (104)	PLG0206 CAMHB ^1^	0.25 to 4
	PLG0206 RPMI ^1^	0.03 to 0.12
	Imipenem	<0.008 to >8
	Levofloxacin	0.12 to >4
	Tigecycline	0.12 to 2
	Vancomycin	1 to 4
	Linezolid	0.5 to >8
	Oxacillin	0.03 to >16
MSSE (46)	PLG0206 CAMHB ^1^	0.25 to 4
	PLG0206 RPMI ^1^	0.03 to 0.12
	Imipenem	<0.008 to 0.06
	Levofloxacin	0.12 to >4
	Tigecycline	0.12 to 2
	Vancomycin	1 to 4
	Linezolid	0.5 to 4
	Oxacillin	0.06 to 0.25
MRSE (58) ^2^	PLG0206 CAMHB ^1^	0.25 to 4
	PLG0206 RPMI ^1^	0.03 to 0.12
	Imipenem	<0.008 to >8
	Levofloxacin	0.12 to >4
	Tigecycline	0.12 to 1
	Vancomycin	1 to 4
	Linezolid	1 to >8
	Oxacillin	0.5 to >16
CoNS, non-*epidermidis* (53) ^3^	PLG0206 CAMHB ^1^	<0.12 to 4
	PLG0206 RPMI ^1^	0.015 to 2
	Imipenem	<0.008 to >8
	Levofloxacin	0.12 to >4
	Tigecycline	0.25 to 2
	Vancomycin	0.5 to 2
	Linezolid	1 to 4
	Oxacillin	0.12 to >16
Enterobacterales (22) ^4^	PLG0206 CAMHB ^1^	1 to >8
	PLG0206 RPMI ^1^	<0.12 to >128
	Imipenem	2 to >8
	Levofloxacin	0.06 to >4
	Tigecycline	0.25 to 4
	Ceftazidime	0.12 to >32
	Colistin	0.06 to >16
	Amikacin	1 to >64
*Pseudomonas aeruginosa* (20)	PLG0206 CAMHB ^1^	8 to >8
	PLG0206 RPMI ^1^	0.5 to 4
	Imipenem	4 to >8
	Levofloxacin	0.5 to >4
	Tigecycline	8 to >16
	Ceftazidime	4 to >32
	Colistin	0.12 to 2
	Amikacin	0.5 to >64
*Acinetobacter baumannii* (24)	PLG0206 CAMHB ^1^	2 to 8
	PLG0206 RPMI ^1^	0.25 to 0.5
	Imipenem	1 to >8
	Levofloxacin	4 to >4
	Tigecycline	1 to 16
	Ceftazidime	>32 to >32
	Colistin	0.12 to >16
	Amikacin	2 to >64

Abbreviations: CAMHB, cation-adjusted Mueller–Hinton broth; CoNS, coagulase-negative staphylococci; MIC, minimum inhibitory concentration; MRSE, methicillin-resistant *S. epidermidis*; MSSE, methicillin-susceptible *S. epidermidis*; NA, not applicable. ^1^ CAMHB was used as the medium for testing all organisms and the RPMI-1640 medium was used for PLG0206 only. Precipitation of PLG0206 at ≥8 µg/mL using CAMHB prevented a full evaluation of its in vitro activity. The solubility of PLG0206 was greatly improved in RPMI-1640 broth, allowing testing up to 32 µg/mL. ^2^ For testing PLG0206 in RPMI, one isolate did not grow; data for PLG0206 RPMI are based on 57 isolates. ^3^ Species breakdown for non-*epidermidis* coagulase-negative *Staphylococcus* spp.: *S. hominis* (10), *S. haemolyticus* (10), *S. warneri* (8)*, S. capitis* (4), *S. simulans* (5), *S. lugdunensis* (4), *S. caprae* (4), *S. saprophyticus* (7), and *S. pettenkoferi* (1). ^4^ Species breakdown for Enterobacterales: *Escherichia coli* (8), *Klebsiella pneumoniae* (8), *Proteus mirabilis* (2), *Enterobacter cloacae* (2), *K. oxytoca* (1), and *Citrobacter freundii* (1).

**Table 2:** Activity of PLG0206 in dPBS (pH 7.0) against *S. aureus* biofilms. Data from Mandell et al., 2020 [35].

PLG0206 Dose, μg/mL	dPBS: pH 7.0, CFU/mL
0	460,000 (141,774)
62	1257 (1510)
125	450 (522)
250	280 (226)
500	233 (188)
1000	417 (492)

Abbreviations: CFU, colony-forming unit; dPBS, diphosphate-buffered saline. Notes: Values represent means (± SD) of triplicate experiments showing remaining adherent *S. aureus* biofilm (CFU/mL) on stainless steel implantation devices after treatment with PLG0206 at the indicated concentrations in different buffered solutions.

**Table 3:** Antibiofilm activity of PLG0206 (1.0 mg/mL) in dPBS at different pH levels against infected PJI implants in a murine model. Data from Mandell et al., 2020 [35].

PJI Implant No.	No Drug, CFU/mL	pH 6.5, CFU/mL	pH 7.0, CFU/mL	pH 7.2, CFU/mL	pH 7.4, CFU/mL
1	287,000	40,000	5000	7000	560
2	933,000	183,000	53,000	33,000	133
3	466,000	1,233,000	3000	6900	5660
4	540,000	266,000	80,000	12,600	8300
5	1,200,000	500,000	30,000	40,000	33,000

Abbreviations: CFU, colony-forming unit; dPBS, diphosphate-buffered saline; PJI, periprosthetic joint infection. Note: Values represent CFU/mL of infected PJI implants after treatment with PLG0206 (1.0 mg/mL) in dPBS at adjusted pH values as indicated. Control PJI implants (no drug) were washed with dPBS at pH 7.0 without PLG0206.

## Data Availability

No new data were created or analyzed in this study. Data sharing is not applicable to this article.

## Patents

Ronald C. Montelaro from the Department of Microbiology and Molecular Genetics, University of Pittsburgh School of Medicine, holds US patent No. 8,071,540 B2 (application No. US12/171,806) on WLBU2 (PLG0206), granted 6 December 2011, which is licensed by Peptilogics.
